# Conformational landscape of the yeast SAGA complex as revealed by cryo-EM

**DOI:** 10.1038/s41598-022-16391-0

**Published:** 2022-07-19

**Authors:** Diana Vasyliuk, Joeseph Felt, Ellen D. Zhong, Bonnie Berger, Joseph H. Davis, Calvin K. Yip

**Affiliations:** 1 Department of Biochemistry and Molecular Biology, Life Sciences Institute, The University of British Columbia, Vancouver, V6T 1Z3 Canada; 2 Computational and Systems Biology, Massachusetts Institute of Technology, Cambridge, MA USA; 3 Computer Science and Artificial Intelligence Laboratory, Massachusetts Institute of Technology, Cambridge, MA USA; 4 Department of Biology, Massachusetts Institute of Technology, Cambridge, MA USA

**Keywords:** Cryoelectron microscopy, Histone post-translational modifications, Biochemistry, Structural biology

## Abstract

Spt-Ada-Gcn5-Acetyltransferase (SAGA) is a conserved multi-subunit complex that activates RNA polymerase II-mediated transcription by acetylating and deubiquitinating nucleosomal histones and by recruiting TATA box binding protein (TBP) to DNA. The prototypical yeast *Saccharomyces cerevisiae* SAGA contains 19 subunits that are organized into Tra1, core, histone acetyltransferase, and deubiquitination modules. Recent cryo-electron microscopy studies have generated high-resolution structural information on the Tra1 and core modules of yeast SAGA. However, the two catalytical modules were poorly resolved due to conformational flexibility of the full assembly. Furthermore, the high sample requirement created a formidable barrier to further structural investigations of SAGA. Here, we report a workflow for isolating/stabilizing yeast SAGA and preparing cryo-EM specimens at low protein concentration using a graphene oxide support layer. With this procedure, we were able to determine a cryo-EM reconstruction of yeast SAGA at 3.1 Å resolution and examine its conformational landscape with the neural network-based algorithm cryoDRGN. Our analysis revealed that SAGA adopts a range of conformations with its HAT module and central core in different orientations relative to Tra1.

## Introduction

A key level of transcriptional regulation in eukaryotic cells occurs at the level of chromatin, the DNA–protein complex that packages genomic DNA within the confine of the nucleus. The fundamental packaging unit of chromatin is the nucleosome, which consists of ~ 147 base pairs of DNA wrapped around an octameric protein complex containing two copies of four core histones (H2A, H2B, H3, H4)^1^. Chromatin prevents transcriptional machinery from accessing the genome, and for this reason, the structure of chromatin needs to be dynamically altered to allow transcription to occur^2^. Post-translational modifications of histones can induce changes to chromatin structure by altering nucleosome stability and/or by acting as signals to recruit other chromatin modifying factors^3^. One of the most extensively studied chromatin modifying factors is the Spt-Ada-Gcn5-Acetyltansferase (SAGA) histone acetyltransferase (HAT) complex. In particular, the yeast *Saccharomyces cerevisiae* SAGA complex has served as a paradigm for understanding the molecular basis of histone modification.

The *S. cerevisiae* SAGA complex consists of 19 core subunits that form a stable assembly of ~ 1.8 MDa in overall mass. Systematic gene deletion combined with mass spectrometry analysis revealed that the SAGA subunits are organized into four modules: histone acetyltransferase (HAT) (Gcn5, Ada2, Ada3, Sgf29), deubiquitination (DUB) (Ubp8, Sgf73, Sgf11, Sus1), core (Spt7, Spt8, Spt3, Spt20, Ada1, Taf5, Taf6, Taf9, Taf10, Taf12), and Tra1 (Tra1)^4–6^. The HAT and DUB modules are catalytic and mediate histone acetylation and deubiquitination, respectively. The catalytic Gcn5 subunit of the HAT module mediates the transfer of an acetyl group from acetyl-CoA to the ε-amino group on distinct lysines of H3^7^. It is thought that histone acetylation, which is correlated with elevated transcription, destabilizes histone-DNA interactions and results in a more open chromatin structure^8,9^. On the other hand, the DUB module catalyzes the removal of ubiquitin on K123 of H2B and enables the recruitment of Ctk1 to the nucleosome, a requirement for advancing transcriptional activation and elongation^10^. SAGA’s Tra1 module contains its largest subunit Tra1 which recruits general transcription factors like Gal4 and Gcn4 to target SAGA to specific genes^11^. Within the core module, SAGA’s TAF subunits are thought to serve a structural role^12^, whereas the Spt3 and Spt8 subunits of this module help regulate transcriptional activation by binding TATA-binding protein (TBP), which is an important component of the preinitiation complex^13–15^. Precisely how the different modules and subunits coordinate with one another in the context of the full SAGA assembly is not fully understood.

Previous negative stain electron microscopy (EM) analysis revealed that yeast SAGA adopts a tri-lobal overall architecture, with an oval-shaped “head” perched on top of a more globular “core” that is connected to an extended “tail”^16^. Subsequent EM-based labeling in conjunction with chemical crosslinking coupled to mass spectrometry studies generated further insights into the subunit organization of SAGA. Specifically, we found that the 433 kDa Tra1 subunit makes up the entire head region, and Spt and Taf subunits constitute the core with the HAT module attaching to the periphery of this core^16,17^. Additionally, SAGA was observed to adopt three major conformations, where the core and tail regions were positioned differently with respect to the head region^17^.

Until recently, high-resolution structural information for SAGA was limited to structures of the full DUB module^18,19^ and three subunits in isolation obtained by X-ray crystallography^20,21^. Advances in cryo-EM technologies have enabled the determination of the high-resolution structures of the large Tra1 subunit^22,23^ and, more recently, the Tra1 and the core modules in the context of full yeast SAGA^24,25^. While these exciting developments laid the foundation to investigate fundamental questions concerning the mechanism-of-action and regulation of SAGA, the large overall size and complex composition of SAGA continue to pose technical challenges to structural investigations. Furthermore, the inherent conformational flexibility of SAGA has thus far precluded visualization of the two catalytic modules within the complex at high resolution. Here, we describe an optimized workflow for purifying native yeast SAGA and preparing cryo-EM specimen of this complex. This procedure not only improves reproducibility of purification and stability of isolated yeast SAGA but also significantly reduces sample requirement for cryo-EM analysis. We were able to determine a cryo-EM reconstruction of yeast SAGA at high resolution using specimens prepared from this workflow and systematically examine the conformational landscape of this dynamic multi-modular complex. Combined with the ease of genetic manipulation of the yeast genome, this workflow could serve as a useful tool for analyzing structure-guided mutants to provide more insights into the mechanism of transcription activation.

## Results and discussion

### Optimizing native yeast SAGA purification and cryo-EM specimen preparation

**Figure 1:**
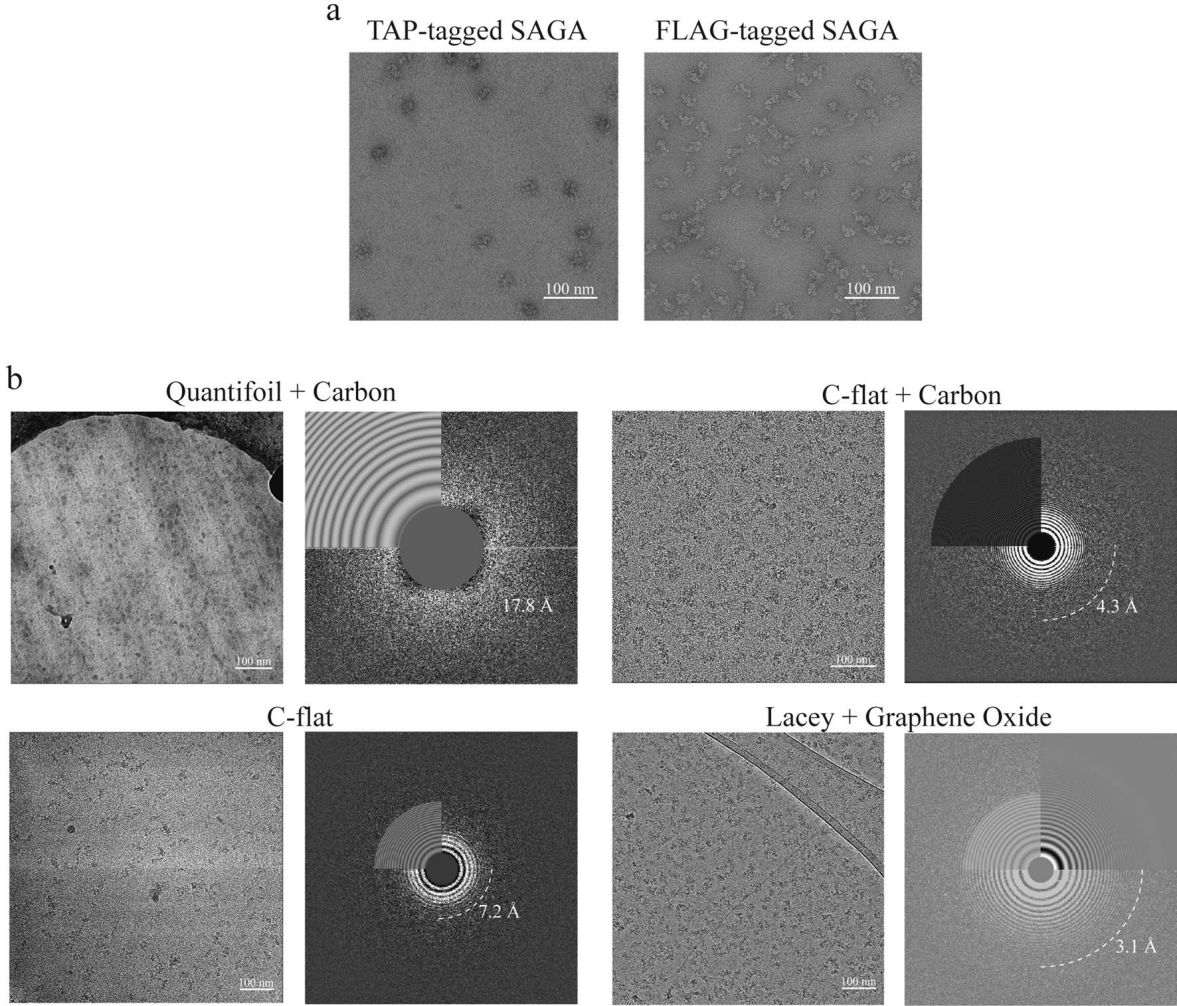
Optimization of *S. cerevisiae* SAGA cryo-EM specimen preparation. (**a**) Representative raw images of negatively stained TAP-purified and FLAG-purified SAGA. (**b**) Representative cryo-EM micrographs of SAGA vitrified on Quantifoil grids with continuous carbon support taken at a defocus value of 3 μm, C-flat carbon grids without a support layer at a 4.5 μm defocus, C-flat grids with carbon support at a 2.5 μm defocus and Lacey carbon grids with graphene oxide support layer at a defocus value of 2.5 μm.

The complex composition of yeast SAGA precluded the use of recombinant expression system for producing and reconstituting this assembly in vitro for biochemical and structural analyses. Previous negative stain EM analysis of budding yeast *Saccharomyces cerevisiae* SAGA by us and others used the conventional two-step tandem affinity purification (TAP) method to isolate native SAGA^16^. The requirement of a lengthy TEV-protease cleavage elution and the need to carry out a second calmodulin-based affinity purification made SAGA prone to dissociation and aggregation. We thus replaced the TAP tag with a 3xFLAG tag and streamlined the purification procedure to a single affinity chromatography step. We also found that the addition of a glycerol gradient with limited glutaraldehyde chemical crosslinking (GraFix) step after anti-FLAG purification improved the quality of the purified complex (Fig. S1)^17^. Negative stain EM analysis of SAGA purified using anti-FLAG in conjunction with GraFix showed that the sample obtained from this procedure contains substantially larger number of intact particles and fewer dissociated complexes and aggregates (Fig. 1a).

Although our revised purification procedure is more effective in preserving the structural integrity of yeast SAGA, the overall yield from a typical purification involving 4L of yeast culture (~ 20–25 g of cell mass) remains relatively low. This, together with the fact that yeast SAGA is prone to aggregation upon sample concentration, made it impossible to obtain the high protein concentration (~ 1 to 2 mg/mL) required for direct vitrification. In the recently reported *S. cerevisiae* SAGA cryo-EM study, the issue of low abundance of native SAGA was resolved by isolating this complex from 100 L of yeast culture^24^. However, growing and harvesting cultures at this scale is not easily accessible to a typical research laboratory and creates barrier to further cryo-EM investigations of yeast SAGA. To overcome this impediment, we explored vitrification using grids containing a support layer that allows for adsorption of particles and thus reduces the sample demand for vitrification. We tested freezing yeast SAGA isolated from 20 to 25 g of cells using our new procedure. We were able to vitrify relatively low concentration of purified SAGA with good distribution of particles. However, the ice thickness and quality differ significantly when different grid types and support layers are used (Fig. 1b). More specifically, Quantifoil grids containing a continuous carbon support film routinely resulted in thick ice. Freezing on C-flat grids with a thin carbon support layer generated thinner and more uniform ice thickness, but the high background noise from the carbon made it impossible to visualize individual particles unless we imaged at high defocus. At the end, we found that lacey carbon grids in combination with graphene oxide support layer provided the best combination for obtaining high-quality vitrified yeast SAGA specimens for cryo-EM analysis. Notably, the variable sized holes in the lacey carbon grids allowed us to obtain thin vitrified specimens. This, together with the negligible background from the graphene oxide support, enable us to unambiguously detect SAGA particles in images taken close to focus (Fig. 1b).

### Graphene oxide support layer facilitates high-resolution cryo-EM analysis of SAGA

Two research groups independently determined the cryo-EM structures of the yeast SAGA core from *S. cerevisiae* and *Pichia pastoria* at high-resolution (~ 3.5 Å) using highly concentrated sample vitrified without a support layer^24,25^. To determine if specimens prepared from our low-yield procedure are suitable for high-resolution structural analysis, we first collected a dataset consisting of 17,955 movies from our vitrified specimens using the K3 direct electron detector on a Titan Krios microscope and a defocus range (− 1.5 to 3.0 μm) similar to that used in previous cryo-EM investigations of yeast SAGA. After motion correction with MotionCor2, well-defined SAGA particles were readily detected, and we were able to extract a total of 1,175,663 particles from template-based autopicking. Subsequent two-dimensional analysis generated class averages with overall quality matching those reported in previous studies using specimens vitrified without a support layer. More specifically, the most highly populated classes show SAGA laying “flat” on a plane with a prominent “head” region containing sufficient details to visualize secondary structures, a central region that connects the head to a “tail” region that have a more “washed out” appearance (Fig. S2). Amongst the different classes, we observed marked differences in the orientations of the core and tail with respect to the position of the head, illustrating the conformational flexibility of the overall assembly.

**Figure 2:**
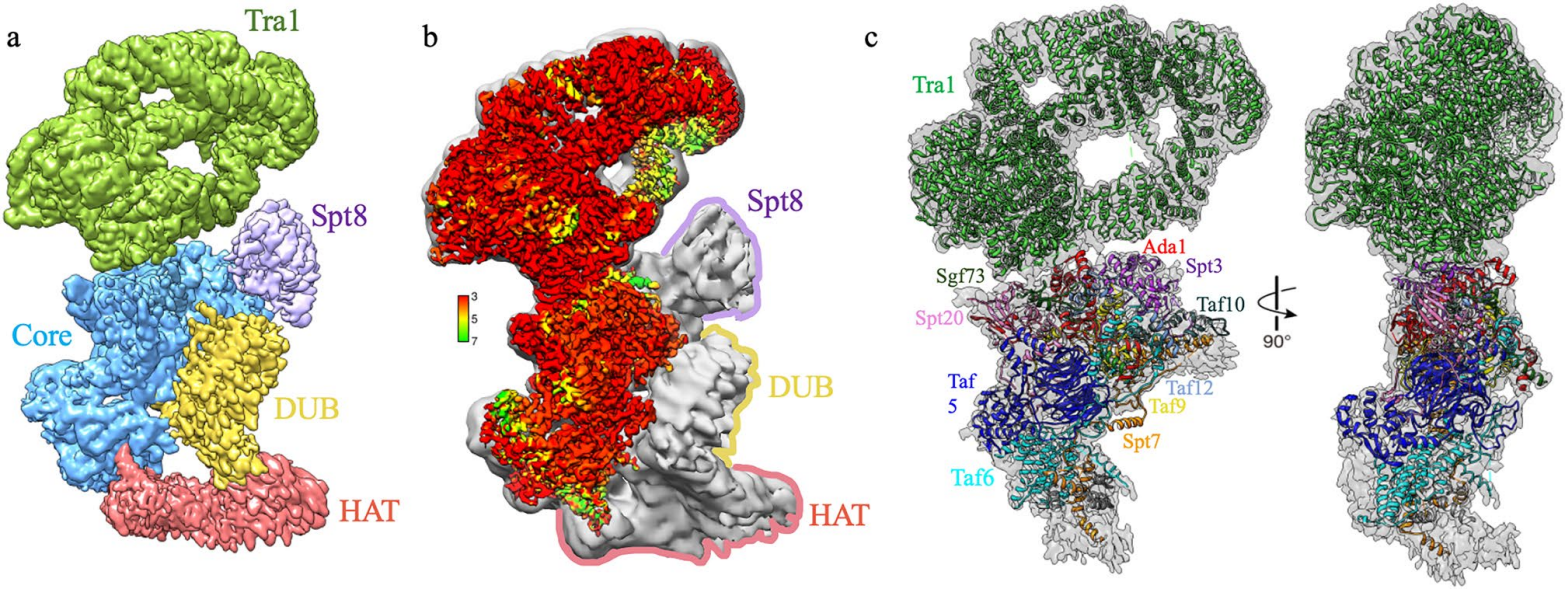
Structure of the *S. cerevisiae* SAGA complex. (**a**) Cryo-EM reconstruction of SAGA. Tra1, core, HAT, DUB modules and Spt8 subunit are indicated by color. The different regions were segmented using UCSF Chimera and verified by fitting the available high-resolution structural model of the Tra1 subunit and the SAGA core module. (**b**) Low-pass-filtered SAGA reconstruction and high-resolution cryo-EM map coloured according to local resolution. (**c**) Front and side views of the SAGA complex with the SAGA cryo-EM structure (displayed as a ribbon model) from Wang et al.^24^ fit into the map. The subunits of Tra1 and core modules are indicated by color. All illustrations were prepared using UCSF Chimera^31^.

**Figure 3:**
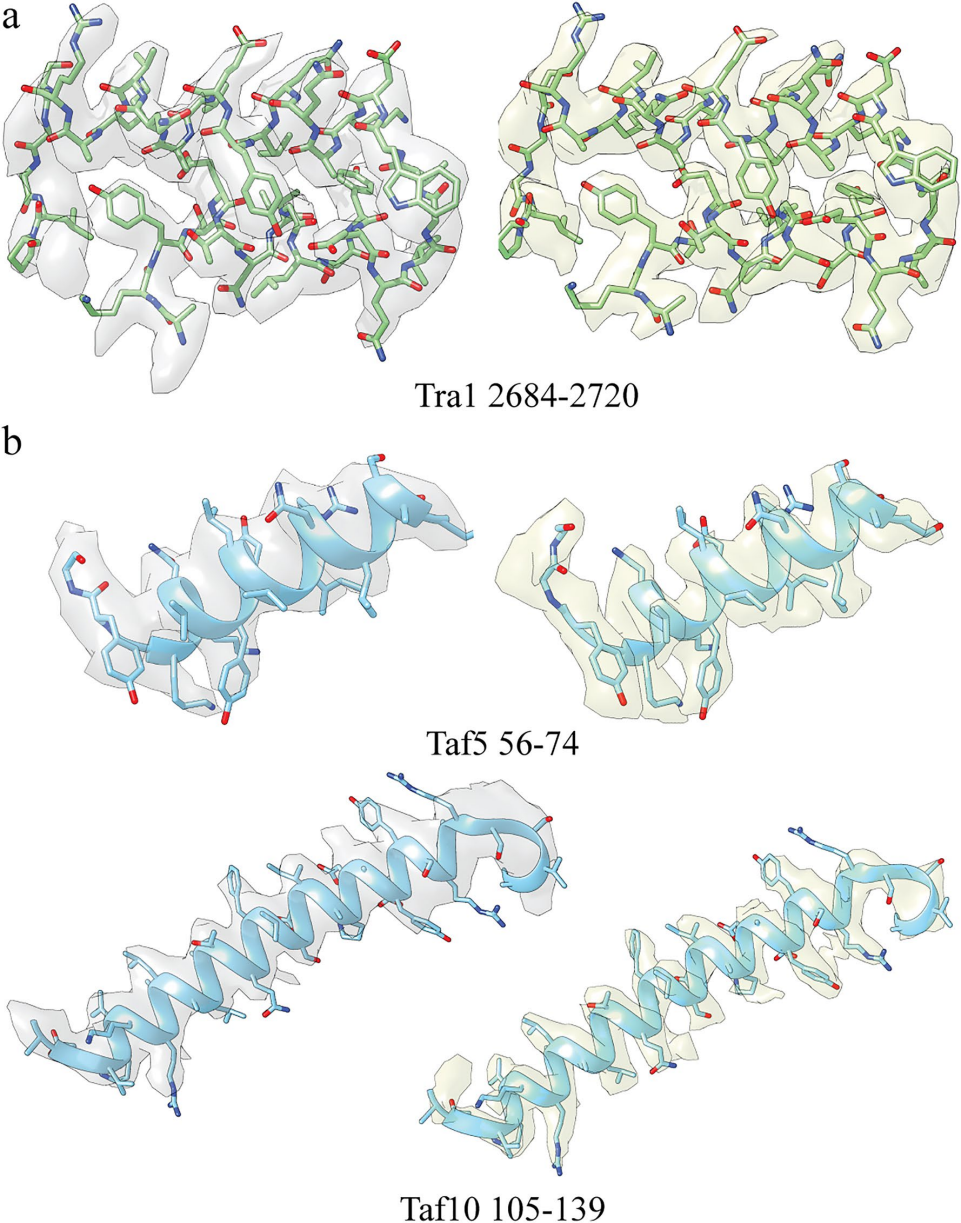
Cryo-EM density analysis of SAGA. (**a**) Cryo-EM density in select regions of Tra1 of the obtained map (left) and the map from Wang et al. (right)^24^. (**b**) Cryo-EM density in select regions of the core module of the obtained map (left) and the map from Wang et al. (right)^24^. All illustrations were prepared using UCSF Chimera^31^.

After multiple rounds of 2D and 3D classification in conjunction with focused refinement, we were able to generate a consensus reconstruction of SAGA at 3.1 Å resolution. Consistent with previous negative stain and cryo-EM investigations, our high-resolution reconstruction contains four modules (Fig. 2a). The prominent head module was resolved to an average local resolution of 2.9 Å (Fig. 2b) and we could visualize side chain densities in many regions of this map (Fig. 3a). We could unambiguously fit the high-resolution structural model of yeast Tra1 derived from previous cryo-EM analysis without adjustments of this model (Fig. 2c), confirming that the Tra1 module is the most rigid and least conformationally flexible module of yeast SAGA.

Directly connected to the head module is the core module, which was resolved to an average location resolution of 3.2 Å (Fig. 2b). Previous cryo-EM studies succeeded in building the atomic models of the core module composed of Spt3, Spt7, Spt20, Taf5, Taf6, Taf9, Taf10, Taf12 and Ada1 subunits^24,25^. We were able to dock this structural ensemble into our map with minimal local adjustments (Fig. 2c) and observe well modeled side chain densities in many regions of the core map (Fig. 3b). By contrast, the HAT and DUB modules and the Spt8 subunit that mediates TBP binding could not be resolved to high-resolution, consistent with prior cryo-EM analyses. This can be attributed to conformational flexibility of these two catalytic modules, which are thought to undergo extensive rearrangement to enable dynamic interaction with the nucleosome substrate. Notably, the DUB module region of our cryo-EM map lacks sufficient resolution to allow accurate fitting of the cognate crystal structure, and the HAT module region of our map did not yield further insights into its subunit organization.

### Analysis by cryoDRGN reveals large scale motions between SAGA modules

The conformational landscape of yeast SAGA and the basis of inter-module movements are poorly understood. Our previous negative stain EM analysis suggested that yeast SAGA adopts three major conformations (“arched”, “curved”, and “donut”) distinguished by the orientation of the mobile “tail” region with respect to the rest of the complex^17^. To assess the flexibility of SAGA and to resolve the conformations at higher resolution, we analyzed SAGA’s conformational landscape in the solution/frozen hydrated state using cryo-EM analysis. We first inspected class averages generated from our 2D analysis of the cryo-EM data (Figs. S2b, S3). Although the majority of these averages show features similar to conformations observed in negative stain analysis: the “arched” conformation where the tail retracts away from the core and causes a sharp kink at the “back” of the core, and the “donut” conformation where the tip of the tail curves up toward the core, they captured SAGA in a greater diversity of conformations. Notably, there are clear differences in the orientation of the core and the tail amongst the different class averages. This indicated that SAGA is capable of undergoing a broader range of motion in solution that has been observed from negative stain analysis.

To analyze the conformations of the SAGA complex in 3D, we employed cryoDRGN, a deep neural network-based algorithm capable of reconstructing a continuous distribution of 3D density maps from heterogeneous cryo-EM datasets^26^. In brief, cryoDRGN simultaneously learns the embedding of individual particle images within a low-dimensional continuous “latent space”, and the generation of 3D density maps from this latent space representation. A major strength of cryoDRGN is that this package learns a nonlinear manifold of structures and does not require specifying initial models or user-defined rigid bodies that place simplifying assumptions on the reconstructed heterogeneity, which can thus result in omission of potentially relevant conformations. Here, we trained an 8-dimensional latent variable model (“Methods”).

**Figure 4:**
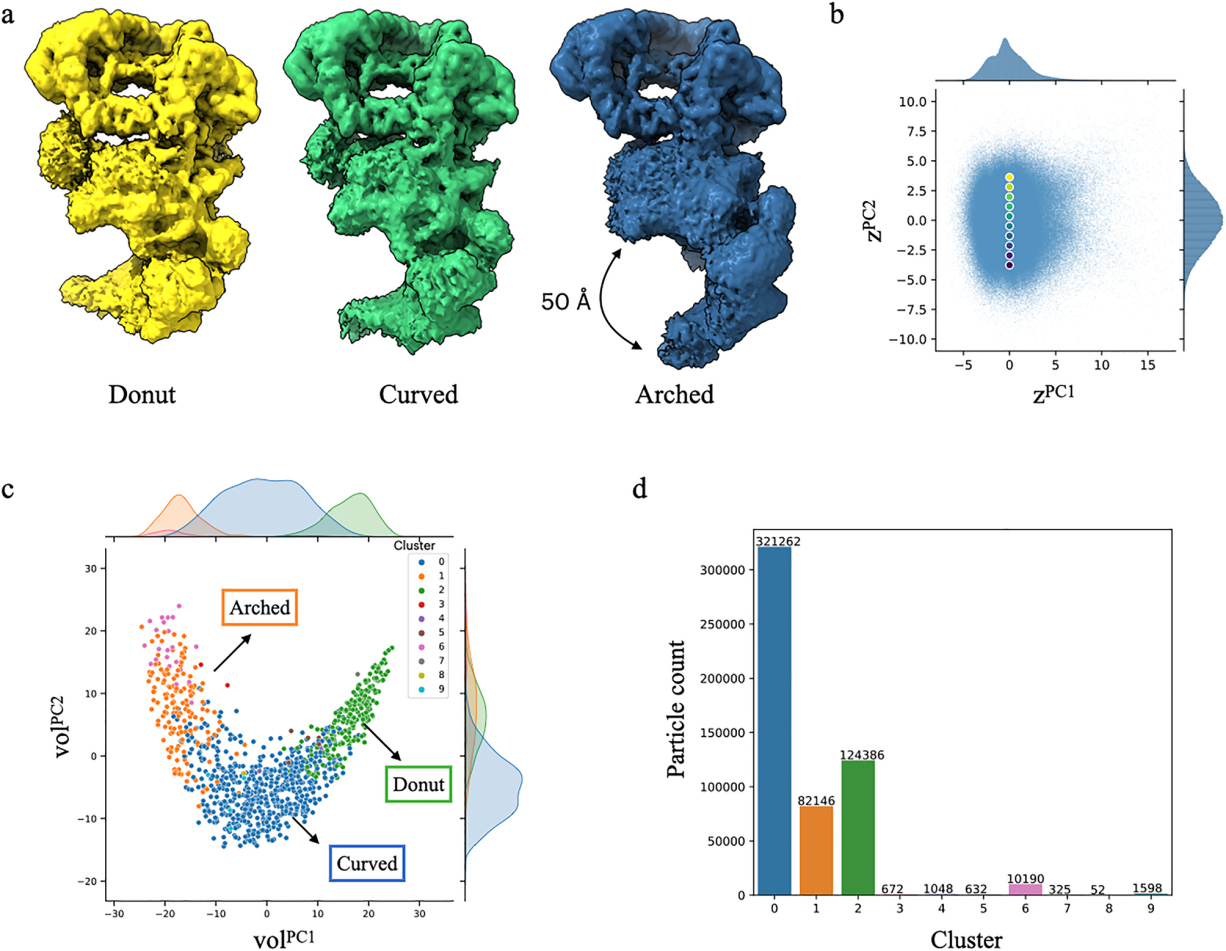
cryoDRGN heterogeneous reconstruction of SAGA. (**a**) CryoDRGN reconstructed density maps of SAGA in “donut”, “curved”, and “arched” conformations. Visualized density maps were generated at the 5th, 50th, and 95th percentile along the principal component (PC) 2 axis of the dataset’s latent embeddings. The view of the SAGA maps is rotated 180° compared to the density maps in Fig. 2a to allow better visualization of the tail region. Note that the prominent DUB module cannot be visualized in this orientation and the TBP density is also slightly obstructed. (**b**) PCA projection of the latent embeddings of SAGA particle images. Points depict a traversal of latent embeddings along the PC2 axis corresponding to density maps in (a) and Supplementary Video S1. (**c**) Clustering of the cryoDRGN volume ensemble. The three largest clusters are labeled according to the associated conformation of SAGA. (**d**) Number of particles in each cluster. All figures were prepared using cryoDRGN^26^.

The ensemble of SAGA 3D reconstructions from different latent space areas revealed unique arrangements of the tail region containing the HAT module (Fig. 4a). The density maps generated from the upper part of the latent space contain particles adopting the “donut” conformation with the tip of the tail positioned closer to the core module. In contrast, the density maps at the bottom part of the latent space contain particles adopting the “arched” conformation with the tip of the tail projecting away from the core (Fig. 4b). Lastly, density maps generated from the center of the latent space are mostly in intermediate “curved” positions, with the tail adopting a slight curvature. Amongst the different conformations, the tail region composed of the HAT module is capable of moving up to 50 Å, supporting the theory that the HAT module and possibly the DUB module need to sample a large space to engage the nucleosomal substrate^24^.

We also observe displacement of the core from the head (Video S1). More specifically, the Spt8 subunit, which is flexibly tethered to the rest of the core, retracts from Tra1 when SAGA adopts the “arched” conformation but gradually moves towards Tra1 as the tail curves upwards. Tra1 is anchored to the core via a hinge involving interactions with Taf12 and Spt20 core subunits. Our finding implied that these hinge contacts are flexible to allow large-scale repositioning of the core relative to the head. Based on the cryo-EM structure of SAGA in complex with TBP, Papai et al. proposed a model in which Spt8 transfers TBP to Spt3 followed by a conformational change that facilitates loading of TBP onto DNA^25^. The dislodging of Spt8 from Tra1 that we observed might represent the conformational change SAGA needs to make to allow DNA to be passed between Tra1 and the core to promote TBP loading. Interestingly, SAGA’s HAT activity has been suggested to facilitate TBP release from SAGA^27^. The coordinated movement of the tail and core might be important for SAGA to disengage from TBP.

We next quantitatively analyzed the distribution of SAGA particles by performing “Landscape Analysis” in cryoDRGN, which carries out a suite of analyses on the trained cryoDRGN model to characterize distinct conformational states and to visualize a conformational landscape (Figs. S4, S5). Our analysis showed that the particles are mainly segmented into three clusters that correspond to the SAGA conformations described above, with approximately 17% of particles in the “arched” conformation with a retracted tail, 23% of particles in the “donut” conformation, and the remainder 60% in the intermediate “curved” conformations (Fig. 4c,d). Homogeneous refinement of these particles in cryoSPARC yielded the same conformations (Fig. S6). However, the resulting maps did not resolve to sufficiently high resolution to allow reliable fitting of known models of individual subunits within these maps. Furthermore, gaining further insights into the molecular basis of these coordinated movements would also necessitate a high-resolution structural model of the HAT module which is currently unavailable.

The observation that most particles adopt intermediate conformations suggests that SAGA likely undergoes continuous conformational changes between two discrete states. The structural plasticity of SAGA likely reflects the need to adapt quickly to interact with different substrates and cofactors during various cellular functions.

## Methods

### Purification of SAGA

Endogenously SAGA was purified through a FLAG affinity purification method. Yeast were grown in 2xYPD liquid media until an OD_600_ of ~ 2.5 was reached, then pelleted and frozen at -80 °C for storage. Pellets were freeze-ground to lyse cells, and the powdered cell matter was stored at − 80 °C. A lysis buffer (40 mM HEPES pH 7.4, 350 mM NaCl, 10% glycerol, 0.1% Tween-20, 1 mM PMSF, 0.1 mM sodium orthovanadate, 2 mM benzamidine, 50 mM NaF) with EDTA-free protease inhibitor cocktail was added to 20–25 g of frozen powdered yeast and rocked at 4 °C until fully thawed and resuspended. Lysate was centrifuged at 4000 rpm at 4 °C for 10 min. The partially cleared supernatant was further clarified at 38,700 rpm at 4 °C for 30 min. Subsequent steps in the purification were performed at 4 °C or on ice. The supernatant was incubated with anti-FLAG M2 Affinity Gel resin (Sigma-Aldrich) for 1 h. The resin was washed first with a high salt and detergent buffer (40 mM HEPES pH 7.4, 350 mM NaCl, 10% glycerol, 0.1% Tween-20) and then resuspended in 1 ml of a low salt, detergent-free buffer (40 mM HEPES pH 7.4, 150 mM NaCl, 10% glycerol) containing 25 µg/ml RNase A and incubated for 15 min. The resin was washed with a low salt, detergent-free buffer before elution of the complex was performed by repeated incubation with FLAG peptide (GenScript). The complex was further purified using an adapted GraFix protocol that utilizes a gradient of glycerol and glutaraldehyde meant to stabilize the complex without promoting aggregation before EM analysis^28^. A Gradient Station was used to prepare a 15–30% glycerol gradient (40 mM HEPES pH 7.4, 150 mM NaCl, 15–30% glycerol, 0.01% Tween-20) with a 0–0.05% glutaraldehyde gradient. FLAG elutions were combined and concentrated with an Amicon Ultra 100 K centrifugation filter (Merck Millipore Ltd.) then loaded onto the GraFix gradient. Gradient was centrifuged at 22,600 rpm at 4 °C for 16 h. Gradient was then fractionated using the Gradient Station fractionator and analysed by SDS-PAGE and negative-stain EM. Peak fractions of GraFix purified SAGA protein were combined, and buffer exchange (40 mM HEPES pH 7.4, 150 mM NaCl, 0.01% Tween-20) was performed using a 40 K MWCO Zeba Spin Desalting Column (Thermo Scientific). Flow-through was concentrated using an Amicon Ultra 100 K centrifugation filter (Merck Millipore Ltd.) and analysed using negative-stain EM to evaluate sample quality.

### Grid preparation and cryo-EM data collection

A thin graphene oxide layer was floated onto Lacey Carbon 300 mesh grids as described previously^29^. The grids were glow discharged for 5 s at 15 mA using a Pelco easiGlow glow-discharger. Aliquots of 3 μl of purified SAGA complex were then applied to the grids. The grids were blotted for 2 s with a blot force of -5 and plunge frozen into liquid ethane using a Vitrobot Mark IV (Thermo Fisher) operated at 4 °C and 100% humidity, and stored in liquid nitrogen for data collection. Grids were screened for particle and ice quality at the UBC High Resolution Macromolecular Cryo-Electron Microscopy (HRMEM) facility using a 200 kV Glacios TEM (Thermo Fisher Scientific) equipped with a Falcon 3EC direct electron detector. For data collection, 17,955 movies were acquired on a 300 kV Titan Krios transmission electron microscope (Thermo Fisher) equipped with a K3 direct electron detector and a BioQuantum K3 energy filter with a slit width of 20 eV (Gatan) at the Pacific Northwest Cryo-EM Center (PNCC). Automated data collection was carried out using SerialEM at a nominal magnification of 64,000 × in super-resolution mode corresponding to a pixel size of 0.666 Å, a dose rate of 50 electrons/Å^2^ over an exposure of 50 frames, and a nominal defocus range of − 1.5 to − 3 μm.

### Image processing

Movie stacks were patch motion-corrected using default settings and Fourier cropped by a factor of 2 using cryoSPARC v3.2^30^. The contrast transfer function (CTF) of the resulting micrographs was estimated using the patch CTF estimation job, and images with poor CTF or ice contamination were discarded. Particles were autopicked using 2D class averages from a previous dataset as templates, yielding 1,175,683 particle images. Particles were then extracted using a box size of 450 pixels. One round of 2D classification was performed to remove deformed particles or false-positive images (such as carbon edges), resulting in 813,702 particle images. After three rounds of 3D classification, classes showing high-resolution features, 542,311 particles in total, were subjected to per-particle local motion correction. The particles were then used to carry out global 3D refinement resulting in a reconstruction with an overall resolution of 3.1 Å based on the Fourier shell correlation (FSC) 0.143 criterion. Local refinements of the Tra1 and core modules were performed using the masked Tra1 and core regions as references to further improve the maps quality. The Tra1 and core modules were resolved at 2.9 Å and 3.2 Å respectively. Auto-sharpening was performed in PHENIX, and local resolutions were estimated using the local resolution estimation tool in cryoSPARC. The cryo-EM models of the Tra1 (PDB: 6t9j) and core modules (PDB:6t9k) were fit into the map using UCSF Chimera^31^.

### cryoDRGN analysis

For heterogeneity analysis, 542,314 particles images were downsampled to an image size of 256 pix and analyzed in cryoDRGN version 1.0 with all default settings unless otherwise noted^26^. A downsampled image stack together with CTF parameters and poses associated with each particle from the cryoSPARC consensus 3D refinement described above were used to train a cryoDRGN 8-dimensional latent variable model for 39 epochs on a single Nvidia V100 graphics processing unit (GPU). The encoder and decoder architectures were set to 3 layers of width 1024 (1024 × 3). After training, the model was analyzed using the “cryodrgn analyze” pipeline, where k-means clustering with k = 20 was performed on the predicted latent embeddings for the dataset, and volumes were generated at the cluster centers using the decoder network. The distribution of latent embeddings was visualized with standard dimensionality reduction techniques, PCA and UMAP. To characterize the continuous distribution of density maps reconstructed by cryoDRGN, the model was analyzed using the "cryodrgn analyze_lanscape" tool with all default settings, including generating 500 volumes, performing PCA analysis to extract conformational coordinates describing the profile, and clustering the volumes into 10 classes to produce summary conformational states. To validate the structures of SAGA in different conformations, particles corresponding to the “arched”, “curved”, and “donut” conformations were extracted and imported into cryoSPARC for homogeneous refinements, resulting in maps at resolution of 4.8 Å, 6.3 Å, and 4.7 Å respectively.

## Supplementary Information

Supplementary Information.

Supplementary Video S1.

## Data Availability

The cryo-EM maps of the complete SAGA complex, the Tra1 module, the core module were deposited in the Electron Microscopy Data Bank (EMDB) under accession codes EMD-26792, EMD-26803, EMD-26804, respectively. All data described in this study are available upon request to the corresponding author.
