# Supplementary material for: Conformational landscape of the yeast SAGA complex as revealed by cryo-EM
Source: Sci Rep. 2022 Jul 19;12:12306. doi: 10.1038/s41598-022-16391-0 (PMC9296673; doi:10.1038/s41598-022-16391-0)
Supplement: Supplementary file 1 — Supplementary Information. [file 41598_2022_16391_MOESM1_ESM.docx]

**Conformational landscape of the yeast SAGA complex as revealed by cryo-EM**

Diana Vasyliuk^1^, Joeseph Felt^1^, Ellen D. Zhong^2,3^, Bonnie Berger^3^, Joseph H. Davis^2,4^, Calvin K. Yip^1^

**Supplementary information**

**Supplementary Figure 1.** Purification of endogenous SAGA from *S. cerevisiae*.

**Supplementary Figure 2.** Cryo-EM analysis and structure determination of SAGA.

**Supplementary Figure 3.** Representative 2D class averages from cryoSPARC corresponding to the “arched” and “donut” SAGA conformations.

**Supplementary Figure 4.** Different embedding representations of SAGA particles from cryoDRGN.

**Supplementary Figure 5.** Mean volumes after clustering the ensemble of volumes in cryoDRGN’s landscape analysis.

**Supplementary Figure 6.** Validation of SAGA conformational states with cryoSPARC.

**Supplementary Table 1.** Cryo-EM data collection and refinement.


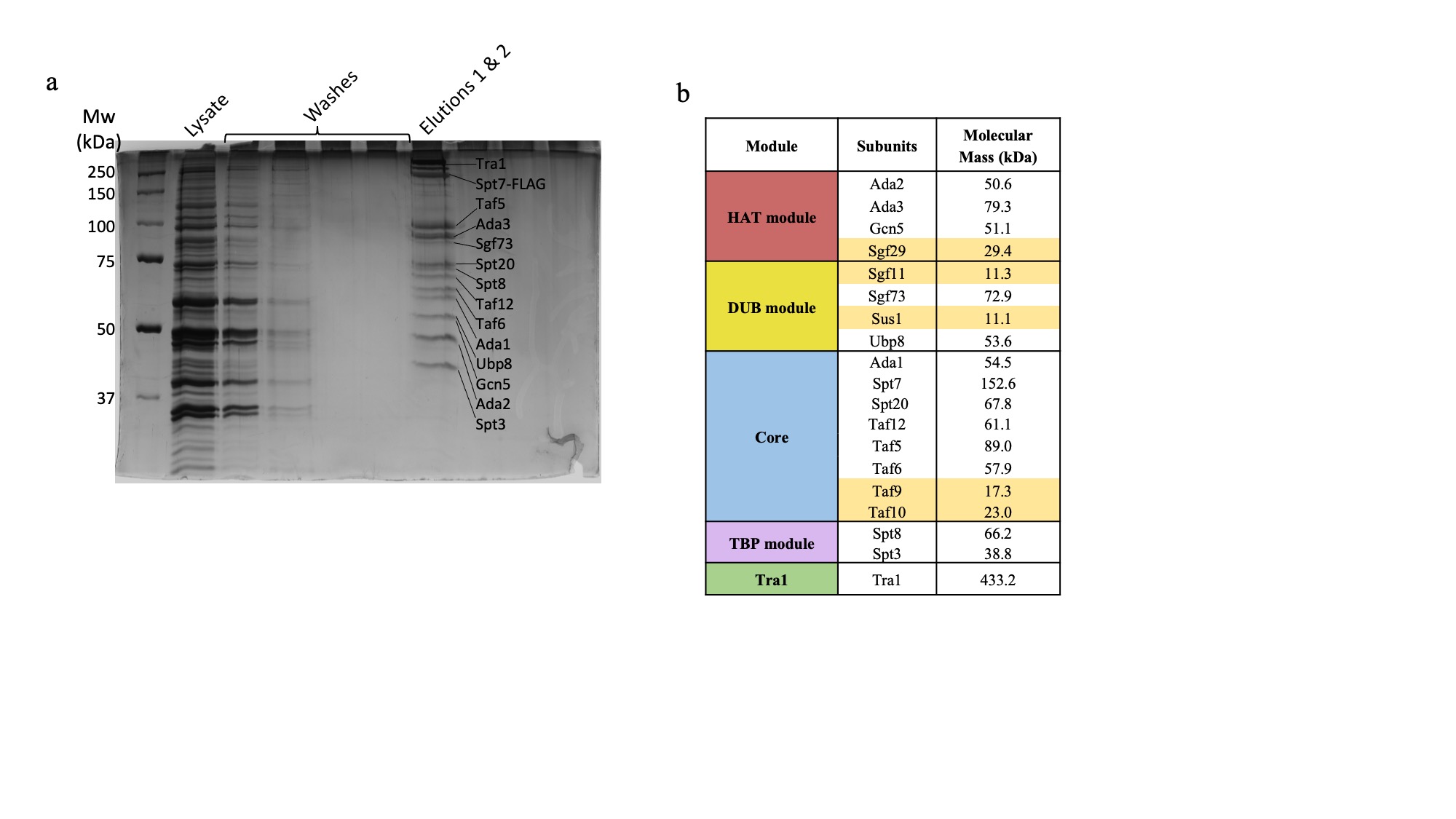


**Supplementary Figure 1. Purification of endogenous SAGA from *S. cerevisiae*. a,** Silver stained SDS-PAGE gel of SAGA subunits after FLAG purification. **b,** Subunit and module organization of SAGA. Subunits highlighted in yellow are not seen on the gel due to low molecular mass.


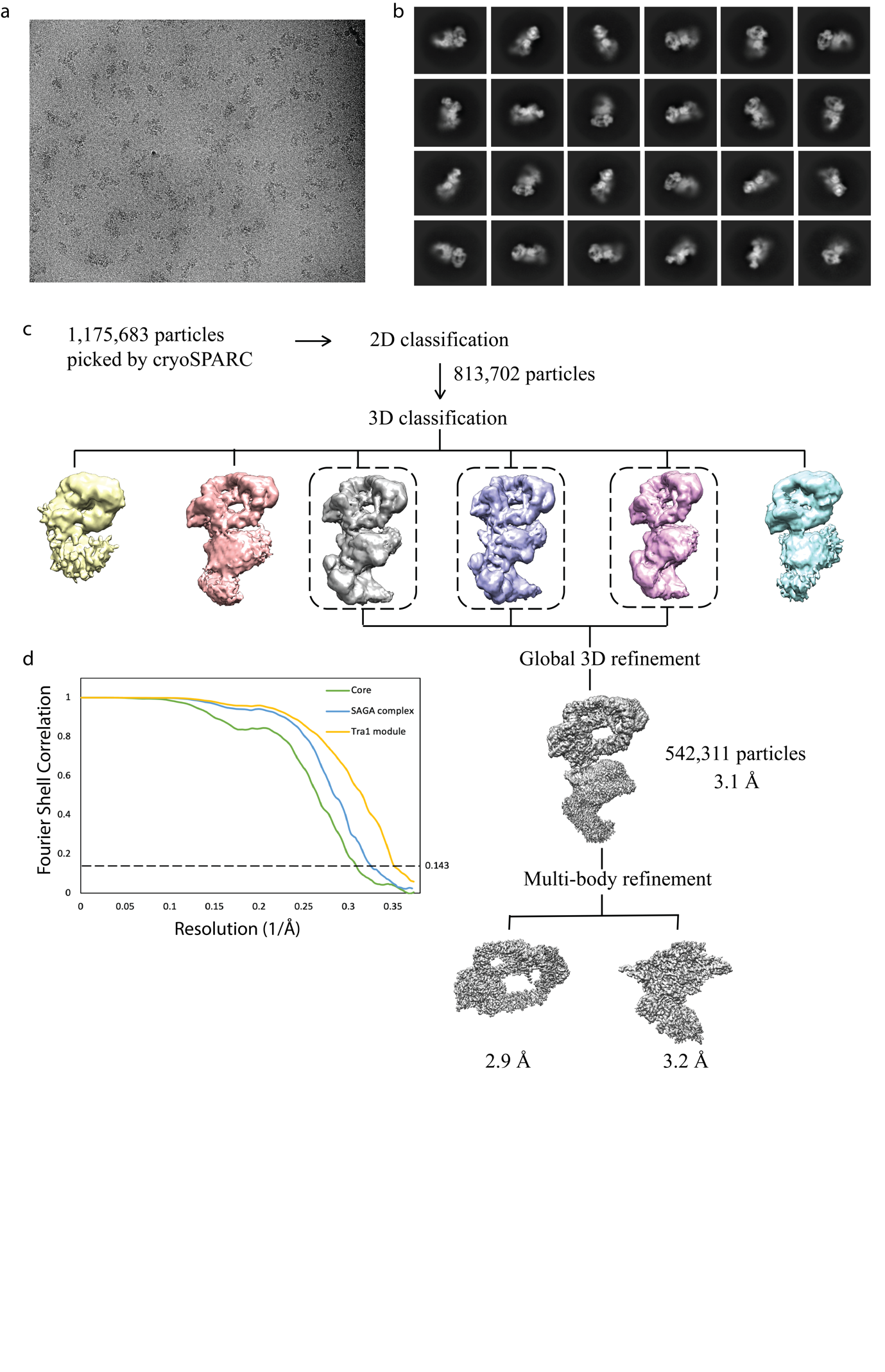


**Supplementary Figure 2.** **Cryo-EM analysis and structure determination of SAGA. a,** Representative cryo-EM micrograph of data collection. **b,** 2D class averages showing high-resolution structural features. **c,** Image processing strategy used to reconstruct SAGA. **d**, FSC curves of the final reconstructions of the entire SAGA complex (blue), the Tra1 module (yellow) and core module (green) obtained from cryoSPARC (30). Resolutions for the gold-standard FSC 0.143 criterion are listed. Cryo-EM maps were displayed using UCSF Chimera (31).


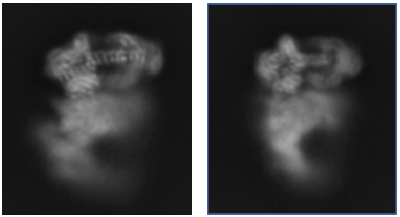


**Supplementary Figure 3. Representative 2D class averages corresponding to the “arched” and “donut” SAGA conformations.** These images were obtained from cryoSPARC (30).

**
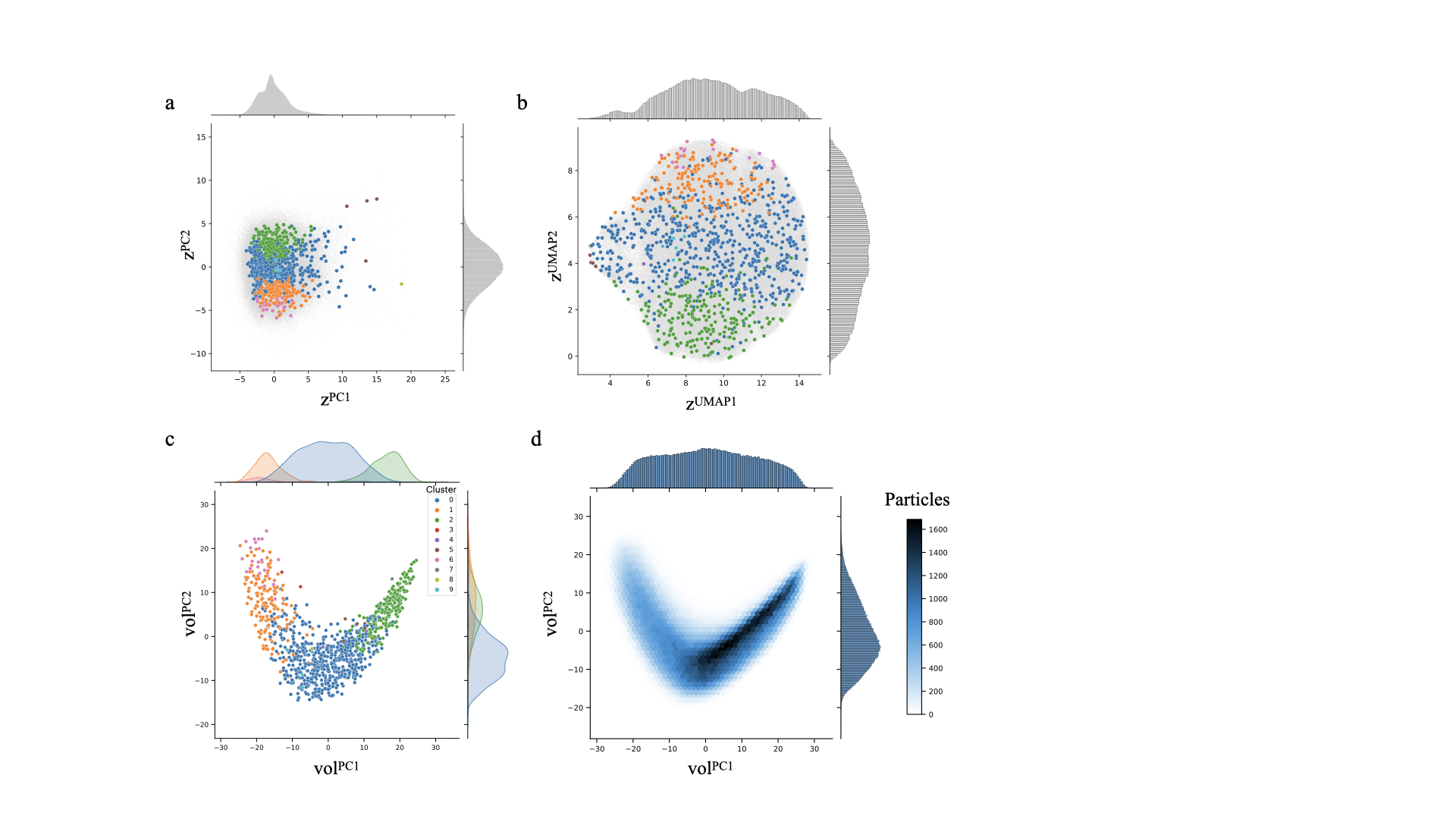
Supplementary Figure 4. Different embedding representations of SAGA particles from cryoDRGN.** Points show the location of 1000 sampled volumes, which are colored by their cluster assignment. **a, b,** Latent variable embeddings (|z| = 8) of SAGA particles visualized in 2D with PCA, a linear dimensionality reduction technique (a), and with Uniform Manifold Approximation and Projection (UMAP), a nonlinear dimensionality reduction technique (b). **c,** Volume-space embeddings of SAGA particles projected along principal components of the sketched volume ensemble. The data manifold follows the continuous transition from the “arched” to the “donut” conformation. Particles at outlying values of the latent representation (e.g. zPC1 > 7.5, zUMAP1 < 5) contain additional density at the periphery of the box potentially due to localization at the air-water interface. A mask is applied around the particle for inferring volume-space embedding in (c). **d,** Mapping of SAGA particles projected along the PCs of the volume ensemble. The different panels were generated by cryoDRGN (26).

**
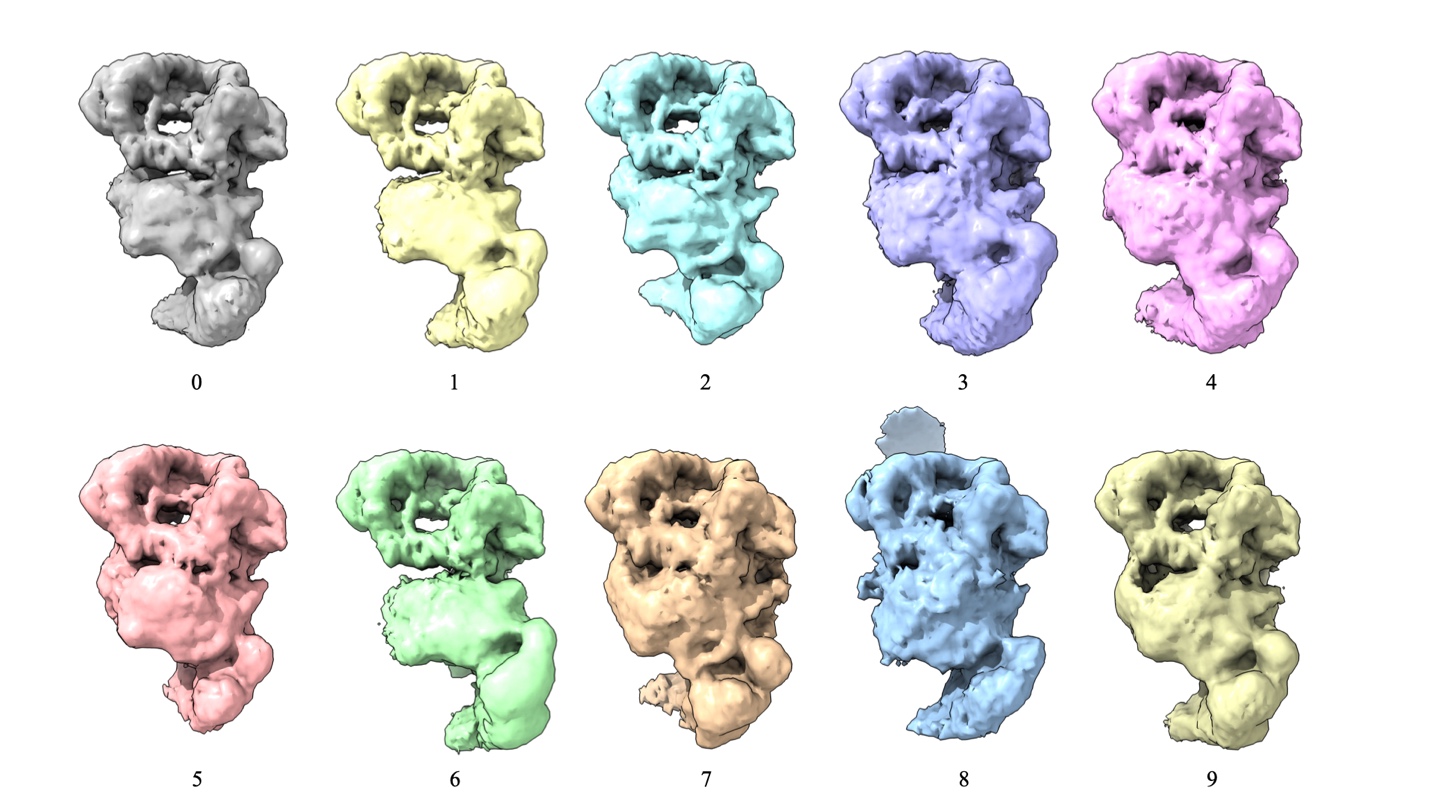
**

**Supplementary Figure 5. Mean volumes after clustering the ensemble of volumes in cryoDRGN’s landscape analysis, viewed at a constant isosurface level.** This ensemble image was generated using UCSF Chimera (31).


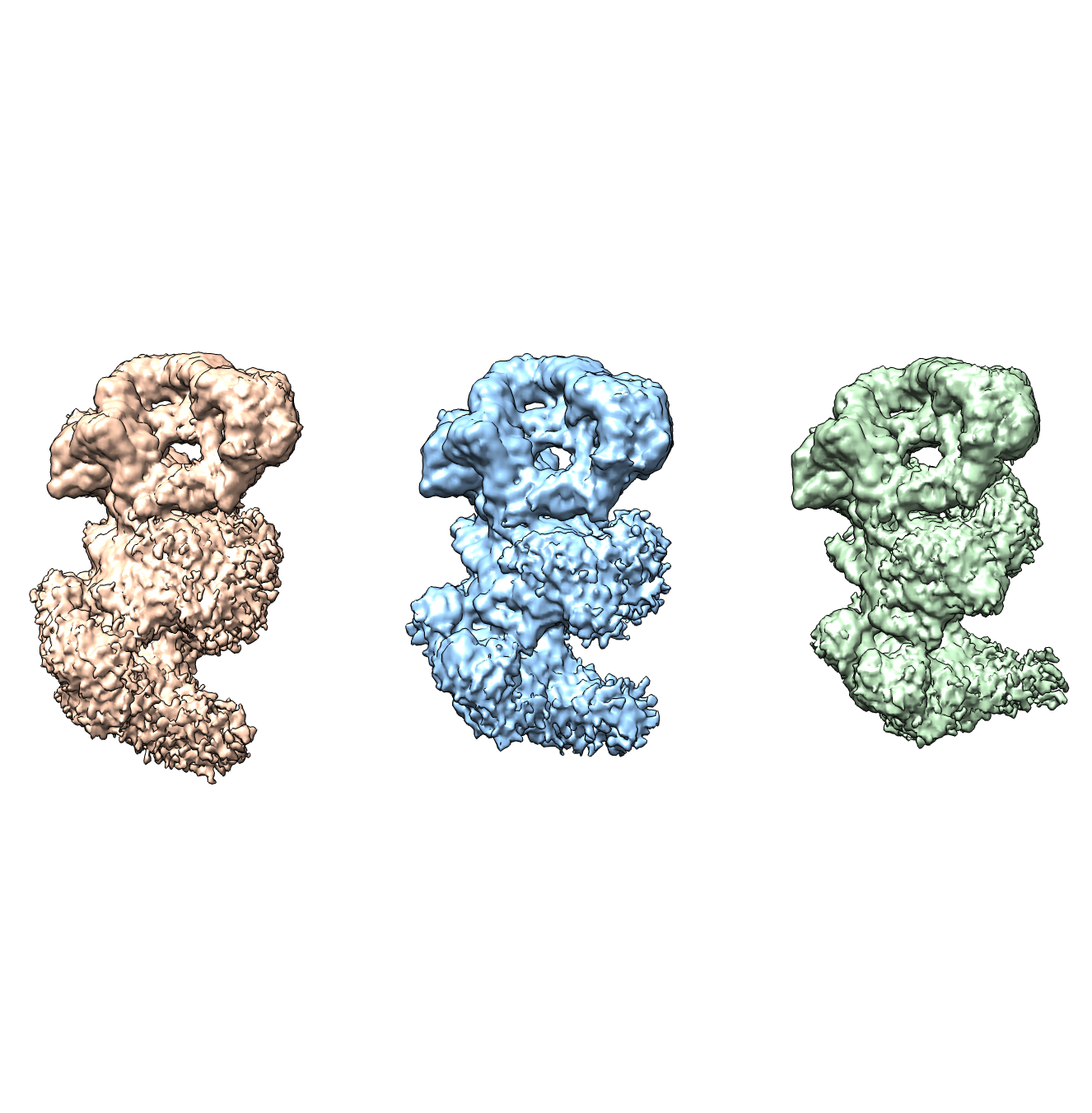


**Supplementary Figure 6. Validation of SAGA conformational states with cryoSPARC.** Density maps from cryoSPARC homogeneous refinement of SAGA in “arched” (orange), intermediate “curved” (blue) and “donut” (green) conformations. These cryo-EM maps were displayed using UCSF Chimera (31).

**Supplementary Table 1. Cryo-EM data collection and refinement**

|  | SAGA | SAGA  Tra1 module | SAGA  core module |
| --- | --- | --- | --- |
| **Data collection and processing** |  |  |  |
| Magnification | 64,000 | 64,000 | 64,000 |
| Voltage (kV) | 300 | 300 | 300 |
| Electron exposure (e/ Å2) | 50 | 50 | 50 |
| Defocus range (μM) | 1.5-3 | 1.5-3 | 1.5-3 |
| Pixel size (Å) | 1.33 | 1.33 | 1.33 |
| Symmetry imposed | C1 | C1 | C1 |
|  |  |  |  |
| Initial particle images (no.) | 1,175,663 | 1,175,663 | 1,175,663 |
| Final particle images (no.) | 542,311 | 542,311 | 542,311 |
| Map resolution (Å) | 3.1 | 2.9 | 3.2 |
| FSC threshold | 0.143 | 0.143 | 0.143 |
| Map resolution range (Å) | 3 – 8.5 | 2.9 – 6.1 | 3.2 – 8.4 |
